# Neuroligin1 Contributes to Neuropathic Pain by Promoting Phosphorylation of Cofilin in Excitatory Neurons

**DOI:** 10.3389/fnmol.2021.640533

**Published:** 2021-02-25

**Authors:** Junlin Ouyang, Xiaping Chen, Shanchun Su, Xiaohui Li, Xueqin Xu, Xinhua Yu, Changbin Ke, Xiaohu Zhu

**Affiliations:** ^1^Department of Orthopedic Rehabilitation, Taihe Hospital, Hubei University of Medicine, Shiyan, China; ^2^Department of Scientific Research, Taihe Hospital, Hubei University of Medicine, Shiyan, China; ^3^Department of Anesthesiology, Institute of Anesthesiology and Pain (IAP), Taihe Hospital, Hubei University of Medicine, Shiyan, China

**Keywords:** neuropathic pain, spinal cord, excitatory neuron, neuroligin1, cofilin, GluR1

## Abstract

Neuropathic pain is a kind of chronic pain that remains difficult to treat due to its complicated underlying mechanisms. Accumulating evidence has indicated that enhanced synaptic plasticity of nociceptive interneurons in the superficial spinal dorsal horn contributes to the development of neuropathic pain. Neuroligin1 (NL1) is a type of excitatory postsynaptic adhesion molecule, which can mediate excitatory synaptic activity, hence promoting neuronal activation. Vglut2 is the most common marker of excitatory glutamatergic neurons. To explore the role of NL1 in excitatory neurons in nociceptive regulation, we used transgenic mice with cre recombinase expression driven by the Vglut2 promoter combined with viral vectors to knockdown the expression of NL1 in excitatory neurons in the spinal dorsal horn. We found that NL1 was upregulated in the L4–L6 spinal dorsal horn in Vglut2-cre^+/–^ mouse subjected to spared nerve injury (SNI). Meanwhile, the expression of phosphorylated cofilin (p-cofilin) and α-amino-3-hydroxy-5-methyl-4-isoxazolepropionic acid receptor subunit 1 (GluR1) was also increased. Spinal microinjection of a cre-dependent NL1-targeting RNAi in Vglut2-cre^+/–^ mouse alleviated the neuropathic pain-induced mechanical hypersensitivity and reduced the increase in p-cofilin and GluR1 caused by SNI. Taken together, NL1 in excitatory neurons regulates neuropathic pain by promoting the SNI-dependent increase in p-cofilin and GluR1 in the spinal dorsal horn. Our study provides a better understanding of the role of NL1 in excitatory neurons, which might represent a possible therapeutic target for alleviating neuropathic pain.

## Introduction

Neuropathic pain is a worldwide problem that can be caused by a lesion or disease of the somatosensory system (Jensen et al., 2011; Colloca et al., 2017; Song et al., 2020). It has been confirmed that central nerve injury and peripheral nerve injury are the two main causes of neuropathic pain (Scholz et al., 2019; Tang et al., 2019). Traumatic nerve injury is the main peripheral mechanism of neuropathic pain. The spared nerve injury (SNI) model is a common animal model of neuropathic pain that makes it possible to study the peripheral mechanisms that occur in sensory processing (Chao et al., 2018; Kumar et al., 2018; Guo et al., 2019). Although some mechanisms of neuropathic pain are well understood, it remains difficult to prevent and treat neuropathic pain without thoroughly elucidating the underlying molecular mechanisms.

Peripheral sensory neurons that are situated in the dorsal root ganglia convey information about noxious and innocuous stimuli to the spinal dorsal horn (Kuner and Flor, 2017; Letellier et al., 2018). Neurons in the spinal dorsal horn can be broadly divided into two types according to their physiological functions: excitatory (glutamatergic) and inhibitory (GABAergic and/or glycinergic) neurons. At present, Vglut2 is considered the most common and reliable marker of excitatory glutamatergic neurons (Wang et al., 2018; Zhang et al., 2018). In dorsal root ganglia neurons, Vglut2 is involved in the development of acute and persistent pain (Rogoz et al., 2012). A previous study determined that EYFP positive dorsal horn neurons are glutamatergic excitatory neurons and mediate nociceptive transmission (Wang et al., 2018).

Neuroligins are postsynaptic and transsynaptic adhesion molecules with a high affinity for presynaptic neuroproteins (Südhof, 2008). Rodents express four neuroligins, which are distributed differently in excitatory and inhibitory synapses (Bemben et al., 2015). Neuroligin1 (NL1) is primarily located in excitatory synapses, and can mediate excitatory synaptic activity, hence promoting neuronal activation (Ye et al., 2017). Although extensive studies have shown that NL1 is associated with pain regulation and hypersensitivity (Guo et al., 2018; Zhao et al., 2018), how NL1 in excitatory neurons participates in nociceptive transmission is not fully known.

Neuroligin1 protein contains an extracellular cholinesterase domain, a transmembrane region, and a cytoplasmic C-terminal domain (CTD). The CTD of NL1 is sufficient to regulate cofilin phosphorylation by activating LIM-domain protein kinase (LIMK) (Liu et al., 2016). Cofilin is an essential protein that binds actin filaments and induces severing and depolymerization, and then effects changes in the actin cytoskeleton and neuron/synapse structure (Davis and Zhong, 2017; Goode et al., 2018). Accumulating evidence has indicated that the expression of phosphorylated cofilin (p-cofilin) is upregulated in neuropathic pain (Valek et al., 2017; Yang et al., 2017; Hu et al., 2019). It is, however, poorly defined whether cofilin is phosphorylated through NL1 in neuropathic pain.

It has been shown that NL1 promotes the differentiation of glutamate synapses by capturing surface-diffusing α-amino-3-hydroxy-5-methyl-4-isoxazolepropionic acid receptors (AMPARs) with a postsynaptic density-95 scaffold (PSD-95) (Shipman et al., 2011; Budreck et al., 2013). A unique intracellular tyrosine of NL1 in hippocampal neurons can regulate AMPAR recruitment during synapse differentiation and potentiation (Letellier et al., 2018). GluR1, an important subunit of AMPARs, is involved in pain hypersensitivity (Guo et al., 2020). We speculated that NL1 might regulate GluR1 recruitment in neuropathic pain.

Neuroligin1 may regulate neuropathic pain differently in excitatory and inhibitory neurons. Thus, in our research, we studied the mechanism by which NL1 in excitatory neurons participates in neuropathic pain. We hypothesized that NL1 might contribute to neuropathic pain by promoting phosphorylation of cofilin and the recruitment of GluR1. To test our hypothesis, we used transgenic mice with Cre recombinase expression driven by the Vglut2 promoter combined with viral vectors to knockdown the expression of NL1 in excitatory neurons in order to specifically examine the effects of NL1 in excitatory neurons in neuropathic pain regulation and the relevant mechanisms.

## Experimental Procedures

### Mice

All surgical and experimental protocols were approved by the Animal Use and Care Committee of Hubei University of Medicine (Shiyan, China) and were performed in accordance with the National Institutes of Health guidelines. VGluT2-IRES-Cre knock-in mice (stock #028863) and Ai3 mice (stock #007903) were obtained from The Jackson Laboratory (Bar Harbor, ME, United States). Wild-type C57BL/6 (WT) mice were provided by the Institute of Laboratory Animal Science, Hubei University of Medicine. The VGluT2::Ai3 mice were generated by crossing male Vglut2-cre^+/+^ mice with female Ai3 mice. The Vglut2-cre^+/–^ mice were generated by crossing male Vglut2-cre^+/+^ mice with female WT mice. The mice were kept in a 12:12 (06:00–18:00) light: dark cycle and were given free access to food and water. Unless otherwise stated, 8–12-week-old male and female mice were used for all experiments. All the studies and tests were conducted between 9 am and 6 pm.

### Spared Nerve Injury Model

As described in previous reports (Decosterd and Woolf, 2000; Shields et al., 2003), Vglut2-cre^+/–^ mice were anesthetized with isoflurane (5% for induction and 2.5% for maintenance) and an incision was made proximal to the lateral side of the right knee to separate the biceps femoris and expose the sciatic nerve and its branches. The common peroneal and tibial nerve branches were ligated with silk suture and about 1 mm of nerve was removed distally, with the sural nerve left intact. After wound closure and post-operative analgesia, the mice were monitored for anxiety symptoms and allowed to recover on a hot mat before returning to their cages. In the sham operation control group, sciatic nerve peroneal nerve and tibial nerve branches were not ligated or cut off, but the operation was performed by the same method.

### Construction of Adeno-Associated Virus (AAV) and Intraspinal Microinjection

Adeno-associated virus vectors with enhanced green fluorescent protein (EGFP) were used to stably knockdown the expression of NL1 in EYFP positive neurons (excitatory neurons). The vector AKD006 (pAKD-CMV-bGlobin-Flex-EGFP-MIR30shRNA) and the NL1 gene (GenBank accession number NM_001357095.1) were recombined by the Obio Technology Company (Shanghai, China). The same vector framework, without gene incorporation but carrying EGFP, was used as the negative control AAV. The viral titer of NL1 was 2.27 × 10^13^ TU/mL. The sh-RNA against Neuroligin1 gene sequence was: 5′-CGAGGCAG TAGGCACAGCGAGAACATTGGGTTCTTTTACATCTGTGG CTTCACTAAAAGAACCCAATGTTCTCGCTTCGCTCACTG TCAACAGCAATATACCTT-3′.

The procedure for microinjection of AAV into the spinal cord was similar to a previous study (Haenraets et al., 2018). The Vglut2-cre^+/–^ mice were first treated with SNI following anesthesia with isoflurane (5% for induction and 2.5% for maintenance) and then immediately subjected to intraspinal AAV injections. The backs were shaved at the dorsal level, and the scraped skin was disinfected with an iodine solution to keep it moist. A longitudinal cut (2.5 cm) was made to expose the vertebral column at the lumbar spinal cord level. Then the lumbar (L4-L6) spinal cord was exposed by tearing or cutting away surrounding muscle and removing tissue remaining on the vertebra or above the dura in the intervertebral space. A glass capillary (25 ± 10 mm diameter) connected to a glass microinjector (10 μL) was used for AAV microinjection, and the needle was inserted 200 μm along the right side of the lumbar spinal dorsal horn midline at a depth of 300 μm to reach the dorsal horn; a target injection volume of 0.3 μL was injected into the mice at a speed of 200 nL/min. After injection, the microsyringe was placed for 5 min to allow the pressure to be balanced, and then the syringe was slowly withdrawn. Then, the skin was sutured and iodine disinfectant was applied. Finally, the mice were put on a hot mat for anesthesia recovery and then transferred to their home cage.

### Behavioral Tests

Mechanical hypersensitivity of the Vglut2-cre^+/–^ mice was assessed using the paw withdrawal threshold (PWT) with a dynamic plantar esthesiometer (Ugo Basile, Comerio, Italy). The ipsilateral and contralateral hind paws of all mice were measured separately between 8:00 a.m. and 11:00 a.m. on days −1, 1, 3, 7, 10, 14, and 21 after corresponding treatments. Before the experiment, each mouse was placed individually in a transparent Perspex box (10 cm × 10 cm × 10 cm) on a wire mesh platform for 30 min in a quiet environment. In each measurement, a straight metal filament (0.5 mm diameter) was raised until it touched the paw of the mouse, and the force (g) was increased until the paw was retracted. The last force on the esthesiometer was the PWT of the mouse. The paw of each mouse was measured once every 10 min for a total of three times, and the average of three values was taken for data analysis.

### Real-Time Polymerase Chain Reaction (PCR)

On day 21 after corresponding treatments, the Vglut2-cre^+/–^ mice were deeply anesthetized with 2% pentobarbital sodium (0.5 mL/100 g, intraperitoneal injection) and the L4–L6 spinal cord was immediately transferred to an ice-chilled lysis buffer through laminectomy. Total RNA was extracted from the spinal cord using Trizol reagent and reverse transcribed according to the manufacturer’s instructions (Takara, Tokyo, Japan). The expression of target genes was analyzed using the ViiA7 Dx system (Applied Biosystems, Carlsbad, CA, United States), with the SYBR Green qPCR Master Mix reagent system (Takara). The forward and reverse primers used in this study were:

GAPDH-F: 5′-GTGAAGGTCGGTGTGAAC-3′
GAPDH-R: 5′-TGAGTGGAGTCATACTGGAA-3′
Neuroligin 1-F: 5′-CCAACAGGAGAACATCGT-3′
Neuroligin 1-R: 5′-AAGCATAACTTCAGGCAATC-3′
Cofilin-F: 5′-GTGTGGCTGTCTCTGATG-3′
Cofilin-R: 5′-GTTCTTCTTGTCCTCACTCA-3′
AMPA 1-F: 5′-GAGCCAATGTGACAGGTT-3′
AMPA 1-R: 5′-TCATAGGTAAGAGCAGAAGTG-3′

### Western Blot

On post-operative day 21, the Vglut2-cre^+/–^ mice were deeply anesthetized with 2% pentobarbital sodium, the L4–L6 spinal cord was quickly removed to an ice-chilled radioimmunoprecipitation assay lysis buffer. The extracted tissues were homogenized in RIPA buffer [20 mM Tris (pH 7.5), 150 mM NaCl, 1% NP40, 0.5% sodium deoxycholate, 1 mM EDTA, and 0.1% sodium dodecyl sulfate (SDS)] supplemented with serine protease and phosphatase inhibitor cocktails (Sigma, United States) for 2 min and disintegrated on ice for 30 min, and the homogenates were centrifuged (12,000 rpm, 15 min, 4°C) to obtain protein. The total protein concentration was determined using a BCA protein assay kit (Thermo Fisher Scientific, Rockford, IL, United States). Subsequently, a western blot assay was conducted as previously described (Laguesse et al., 2017). Tissue homogenates were separated by 10% SDS-PAGE and transferred onto nitrocellulose membrane at 300 mA for 2 h. Membranes were incubated with a blocking solution (5% milk-PBS, 0.1% Tween 20) at room temperature for 30 min and then probed with primary antibodies diluted in blocking solution overnight at 4°C. The primary antibodies were diluted and used in this study were as follows: sheep antineuroligin1 (1:1000, AF4340, R&D Systems), rabbit anti-phospho-cofilin (1:1000, 3313, Cell Signaling Technology), mouse anti-cofilin (1:1000, ab42824, Abcam), rabbit antiGluR1 (1:1000, AB1504, Millipore), rabbit anti-GAPDH (1:1000, 2118, Cell Signaling Technology). Membranes were washed and probed with HRP-conjugated secondary antibodies for 1 h at room temperature. Membrane were developed using ECL and band intensities were quantified using Image Lab software (Bio-Rad, ChemiDoc XRS+).

### Immunofluorescence

Mice were anesthetized with 2% pentobarbital sodium and sequentially perfused with ice-cold phosphate buffered saline (PBS) and 4% paraformaldehyde on day 21 after corresponding treatments. The L4–L6 spinal cords were rapidly removed from the mice, post-fixed in 4% paraformaldehyde for 2 h at 4°C, and then transferred to a 30% sucrose solution diluted with PBS for 48 h at 4°C. The spinal cord was embedded with O.C.T compound at −20°C and then cut into transverse slices of 25 μm thickness using a freezing microtome (Leica, Germany). After being washed with PBS three times, the slices were blocked with 5% normal donkey serum in PBS for 10 min at room temperature and then incubated with primary antibodies overnight at 4°C. The next day, the slices were removed from the refrigerator for 30 min and the slices were washed with PBS three times and incubated with secondary antibodies in darkness for 40 min at 37°C. Finally, after mounting with DAPI (Beyotime, China), the immunofluorescence was detected using a confocal laser microscope (Leica TCS SP8, Wetzlar, Germany).

The primary antibodies were diluted in PBS and used in this study were as follows: sheep antineuroligin1 (1:50, AF4340, R&D Systems), rabbit anti-phospho-cofilin (1:100, 3313, Cell Signaling Technology), rabbit GluR1 (1:50, AB1504, Millipore), and guinea pig antiMAP2 (1:200, 188004, Synaptic System).

The secondary antibodies were diluted in PBS and used in this study were as follows: Alexa Fluor 647 conjugated donkey anti-sheep IgG H&L (1:200, ab150179, Abcam), Alexa Fluor 568 conjugate donkey anti-rabbit IgG H&L (1:200, ab175470, Abcam), and Alexa Fluor 647 conjugated donkey anti-guinea pig IgG H&L (1:200, ab150187, Abcam).

### Statistical Analysis

SPSS 22 software (SPSS, Inc., Chicago, IL, United States) was employed in our study for statistical analysis, and results are expressed as mean ± standard error of the mean (SEM). Analysis of the time-course of SNI-induced tactile allodynia between groups was performed using two-way (group and time) repeated measures analysis of Variane (ANOVA). For all other experiments, differences were compared using one-way ANOVA. Results with a *p* < 0.05 were considered statistically significant.

## Results

### Most Excitatory Neurons Express NL1

**FIGURE 1:**
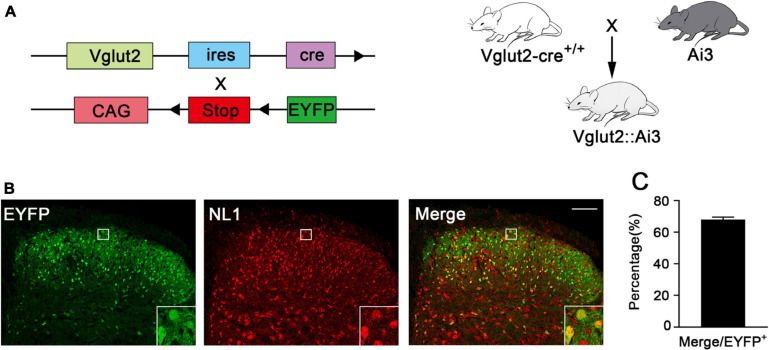
Expression of NL1 in EYFP positive neurons of the spinal dorsal horn **(A)** Schematic diagram for Vglut2-cre^+/+^ and Ai3 reporter sequences. Vglut2-cre^+/+^ mice were crossed with Ai3 mice to generate Vglut2::Ai3 mice. **(B)** Representative confocal images of EYFP positive neurons (green) and NL1 (red) in the right spinal dorsal horn of normal Vglut2::Ai3 mice. Scale bar: 70 μm. **(C)** Percentage of EYFP positive neurons expressing NL1 in total EYFP positive neurons (*n* = 3; Mean ± SEM). The white square region shows NL1 expression in the EYFP positive neurons of the spinal dorsal horn.

To investigate the expression of NL1 in the excitatory neurons of the spinal cord, we crossed Vglut2-cre^+/+^ mice with Ai3 mice to obtain Vglut2::Ai3 mice (Figure 1A), in which the Vglut2 positive neuron would express EYFP under the control of the Vglut2 promoter, thus all of the excitatory neurons in the never system would be labeled with yellow fluorescence. Then we examined the expression of NL1 in the lumbar (L4–L6) spinal dorsal horn of normal Vglut2::Ai3 mice by immunofluorescence labeling with an anti-NL1 antibody. Confocal microscopy images (Figure 1B) showed that excitatory neurons were most abundant in the superficial dorsal horn, and 68 ± 2.6% excitatory neurons expressed NL1 (Figure 1C).

### Upregulation of NL1 in the Spinal Dorsal Horn Promotes the Development of Neuropathic Pain

**FIGURE 2:**
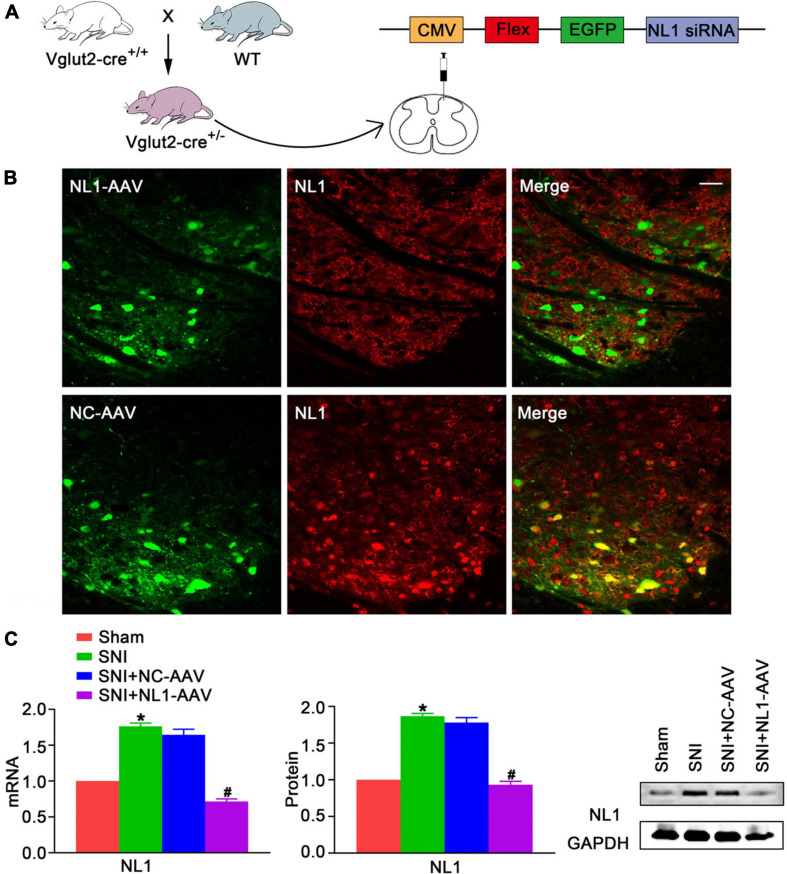
Upregulation of NL1 in the spinal dorsal horn of Vglut2-cre^+/–^ mice following SNI **(A)** Vglut2-cre^+/+^ mice were crossed with WT mice to generate Vglut2-cre^+/–^ mice. NC-AAV or NLI-AAV were injected into the spinal dorsal horn of normal Vglut2-cre^+/–^ mice. **(B)** On day 21 after intraspinal AAV injections, double immunofluorescence staining with AAV (green) and anti-NL1 (red) showing effect of AAV transfection in the right spinal dorsal horn of Vglut2-cre^+/–^ mice. Scale bar: 25 μm. **(C)** Quantitative analysis of NL1 by real-time PCR and western blot in spinal cords of sham, SNI, SNI+NC-AAV, and SNI+NL1-AAV group on post-operative day 21 (*n* = 4; Mean ± SEM; *p < 0.05 vs. Sham group; ^#^*p* < 0.05 vs. SNI+NC-AAV group). SNI, spared nerve injury; SNI mice, mice subjected to SNI; SNI+NC-AAV mice, SNI mice treated with NC-AAV; SNI+NL1-AAV mice, SNI mice treated with NL1-AAV.

To explore whether NL1 was involved in the development of neuropathic pain, the peroneal and tibial nerves were tightly ligated and cut, whilst leaving the sural nerve intact, to establish the SNI model, and the mRNA and protein levels of NL1 in the sham and SNI groups was measured. Real-time PCR (Figure 2C) and western blot analysis showed that both mRNA and protein levels of NL1 were substantially higher in the SNI group (*p* < 0.05) than in the sham group. To further investigate the role of NL1 in excitatory neurons in neuropathic pain, native control adeno-associated virus (NC-AAV) or recombinant RNAi adeno-associated virus targeting NL1 (NLI-AAV) were injected into the lumbar spinal dorsal horn of Vglut2-cre^+/–^ mice after SNI surgery (Figure 2A). To test the effect of AAV transfection, immunofluorescence staining with anti-NL1 was carried out on AAV-positive lumbar spinal cord slices (Figure 2B). Confocal images showed that specific knockdown of NL1 in excitatory glutamatergic neurons. Real-time PCR (Figure 2C) and western blot analysis showed that both mRNA and protein levels of NL1 were remarkably downregulated by NL1-AAV (*p* < 0.05), but not by NC-AAV.

**FIGURE 3:**
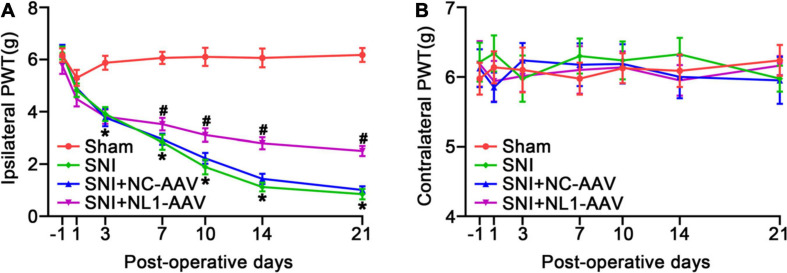
Knockdown of NL1 in EYFP positive neurons reduces the SNI-induced pain hypersensitivity in Vglut2-cre^+/–^mice The PWT was evaluated in the Sham, SNI, SNI+NC-AAV, and SNI+NL1-AAV Vglut2-cre^+/–^ mice on post-operative days –1, 1, 3, 7, 10, 14, and 21. **(A)** SNI induced a decrease in PWT and knockdown of NL1 in EYFP positive neurons attenuated SNI-induced pain hypersensitivity. **(B)** No significant difference was observed on the contralateral side in the four groups (*n* = 8; Mean ± SEM; *p < 0.05 vs. Sham group; ^#^*p* < 0.05 vs. SNI+NC-AAV group). PWT, paw withdrawal threshold. Post-operative days: days after corresponding treatment for four groups of mice. Intraspinal AAV injections were performed immediately after SNI-surgery.

We measured pain thresholds of ipsilateral and contralateral paws in the four groups of mice on post-operative days −1, 1, 3, 7, 10, 14, and 21. The results demonstrated that all mice had the same pre-operative PWT. There were no significant differences on the contralateral side among the four groups (Figure 3B). From day 3, the PWT of the SNI group was significantly decreased compared with the sham group (*p* < 0.05) on the ipsilateral side (Figure 3A). From day 7, neuropathic pain was alleviated by treatment with NL1-AAV compared with NC-AAV treatment (*p* < 0.05). All of these results suggest that NL1 in excitatory neurons plays an important role in the development of neuropathic pain.

### Reduction of NL1 in Excitatory Neurons Impairs the Increase of p-Cofilin in the Spinal Dorsal Horn Following SNI

**FIGURE 4:**
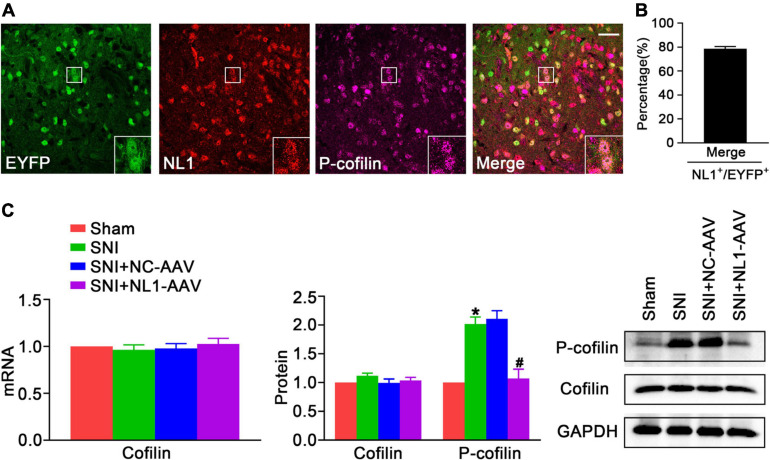
The decrease of NL1 in EYFP positive neurons blocks the SNI-dependent increase of p-cofilin in the spinal dorsal horn of Vglut2-cre^+/–^ mice following SNI **(A)** Representative confocal images of EYFP positive neurons (green), NL1 (red), and p-cofilin (rose red) in the right spinal dorsal horn of normal Vglut2::Ai3 mice. The white square region shows co-localization of NL1 and p-cofilin in EYFP positive neurons. Scale bar: 15 μm. **(B)** Percentage of EYFP neurons expressing NL1 and p-cofilin in EYFP neurons expressing NL1 (*n* = 3; Mean ± SEM). **(C)** Quantitative analysis of cofilin by real-time PCR in spinal cords of sham, SNI, SNI+NC-AAV, and SNI+NL1-AAV Vglut2-cre^+/–^ mice, and quantitative analysis of cofilin and p-cofilin by western blot in spinal cords from the four groups on post-operative day 21 (*n* = 4; Mean ± SEM; *p < 0.05 vs. Sham group; ^#^*p* < 0.05 vs. SNI+NC-AAV group).

Activating the RhoA/LIM kinase/cofilin pathway results in chronic neuropathic pain (Qiu et al., 2016). It has been previously shown that NL1 is sufficient to induce cofilin phosphorylation (Liu et al., 2016). To further analyze whether NL1 in excitatory neurons promotes the development of neuropathic pain by regulation of the phosphorylation of cofilin, lumbar spinal cord slices of normal Vglut2::Ai3 mice were co-stained with antibodies against NL1 and p-cofilin. Confocal images (Figure 4A) showed that p-cofilin was co-localized with NL1 in excitatory neurons in the lumbar spinal cord. 78.6 ± 3.2% of NL1-expressing excitatory neurons expressed p-cofilin (Figure 4B). Real-time PCR (Figure 4C) and western blot analysis showed that both mRNA and protein levels of cofilin were not markedly different in the four groups, while the protein level of p-cofilin was obviously increased in the SNI group compared with the sham group (*p* < 0.05). The increase in p-cofilin following SNI was significantly reduced by treatment with NL1-AAV compared with NC-AAV (*p* < 0.05). These results indicate that the upregulation of p-cofilin might promote the development of SNI, and NL1 in excitatory neurons could regulate cofilin phosphorylation.

### NL1 in Excitatory Neurons Promotes Expression of GluR1 Following SNI

**FIGURE 5:**
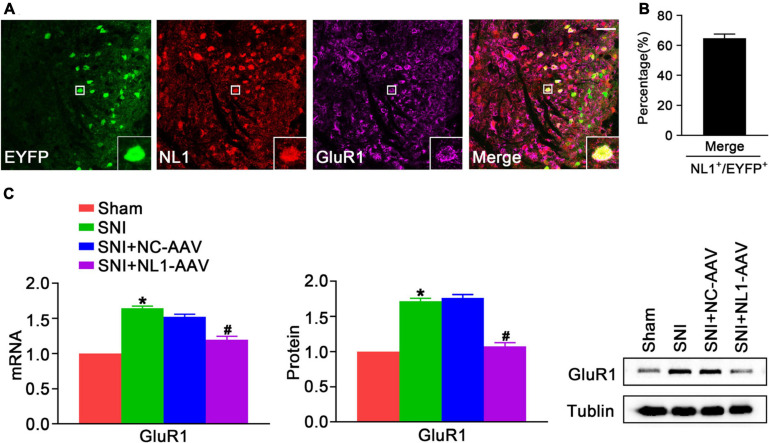
NL1 in EYFP positive neurons promotes expression of GluR1 in the spinal dorsal horn of Vglut2-cre^+/–^ mice following SNI **(A)** Representative confocal images of EYFP positive neurons (green), NL1 (red), and GluR1 (rose red) in the right spinal dorsal horn of normal Vglut2::Ai3 mice. The white square region shows co-localization of NL1 and GluR1 in EYFP positive neurons. Scale bar: 15 μm. **(B)** Percentage of EYFP neurons expressing NL1 and GluR1 in EYFP neurons expressing NL1 (*n* = 3; Mean ± SEM). **(C)** Quantitative analysis of GluR1 by real-time PCR and western blot in spinal cords of sham, SNI, SNI+NC-AAV, and SNI+NL1-AAV Vglut2-cre^+/–^ mice on post-operative day 21 (*n* = 4; Mean ± SEM; *p < 0.05 vs. Sham group; ^#^*p* < 0.05 vs. SNI+NC-AAV group).

GluR1 is a significant subtype of the AMPARs, which are involved in pain hypersensitivity (Guo et al., 2020). Knockout of NL1 in newborn mice leads to a reduction in AMPAR levels at synapses and AMPAR-dependent synaptic transmission in hippocampal slices (Mondin et al., 2011). To explore whether NL1 in excitatory neurons can also facilitate expression of GluR1 and ultimately contribute to the development of neuropathic pain, double staining of NL1 and GluR1 was used to show colocalization in spinal cord slices of normal Vglut2::Ai3 mice, and NL1-AAV was used to knockdown NL1 in excitatory neurons in the dorsal horn of Vglut2-cre^+/–^ mice. Confocal images (Figure 5A) showed that NL1 and GluR1 were colocalized in excitatory neurons in the lumbar spinal cord. 64.7 ± 5.0% of NL1-expressing excitatory neurons expressed GluR1 (Figure 5B). Real-time PCR (Figure 5C) and western blot analysis suggested that the expression of GluR1 was remarkably increased in mice subjected to SNI as compared with sham-operated mice (*p* < 0.05), and this was substantially reversed by NL1-AAV (*p* < 0.05) but not by NC-AAV. These results show that NL1 in excitatory neurons facilitates the expression of GluR1 in neuropathic pain.

## Discussion

In the spinal dorsal horn, about a third of the total neurons are inhibitory GABAergic and glycinergic interneurons (Todd et al., 2003; Foster et al., 2015). Wang et al. (2018) have determined that EYFP positive dorsal horn neurons are glutamatergic excitatory neurons and mediate nociceptive transmission. In our study, we mainly wanted to know the peripheral mechanisms by which some of the proteins expressed in excitatory neurons are involved in neuropathic pain.

Neuroligin1 is an excitatory postsynaptic adhesion molecule (Ye et al., 2017). Reduction of NL1 levels normalizes the increased excitatory synaptic activity and reverses inflammatory pain hypersensitivity (Khoutorsky et al., 2015). Activity-dependent synaptic recruitment of NL1 in the spinal dorsal horn contributes to inflammatory pain (Zhao et al., 2018). Downregulation of spinal NL1 expression ameliorates post-operative pain (Guo et al., 2018). Because it has not been confirmed how NL1 plays a key role in neuropathic pain, how NL1 in excitatory neurons is involved in neuropathic pain has not been investigated either. We wanted to learn about the mechanism by which NL1 is involved in neuropathic pain. Our research found that excitatory neurons were most abundant in the superficial dorsal horn, that most excitatory neurons expressed NL1, that NL1 was upregulated in Vglut2^+/–^ mice after SNI, and that blocking NL1 in excitatory neurons in the spinal dorsal horn by RNAi AAV increased the PWT of mice subjected to SNI. These results indicated that NL1 in excitatory neurons participates in the induction and maintenance of neuropathic pain.

In a similar model of neuropathic pain, Lin et al. (2015) found no upregulation of NL1 expression but an increased co-expression of NL1 with PSD95. We used different animals and models, which may be two main reasons for the different results. They used adult male Sprague-Dawley rats (200–250 g) and spinal nerve ligation model, in which the L5–L6 spinal nerves of rats were dissected and ligated. And we used male 8–12-week-old Vglut2-cre^+/–^ mice and spared nerve injury model, in which the common peroneal and tibial nerve branches of mice were ligated and about 1 mm of nerve was removed distally, with the sural nerve left intact.

Hu et al. (2019) have shown that inflammatory pain is accompanied by a reduction in LIMK / cofilin phosphorylation and actin polymerization. Yang et al. (2017) have found that SNI causes a significant increase in p-cofilin levels. Qiu et al. (2016) have demonstrated that inhibiting cofilin phosphorylation attenuates neuropathic pain. Our results also suggested that p-cofilin was markedly upregulated in Vglut2-cre^+/–^ mice subjected to SNI, but the level of cofilin was not changed significantly. Thus, these current studies illustrate that p-cofilin is linked to the SNI-dependent increase in neuropathic pain.

Liu et al. (2016) have indicated that NL1 induces cofilin phosphorylation and regulates spine / synaptic plasticity via activation of Rap1/LIMK1-mediated actin reorganization. We wanted to further investigate whether NL1 in excitatory neurons contributes to the development of neuropathic pain by inducing cofilin phosphorylation. Our study found that NL1 and p-cofilin were co-localized in excitatory neurons of the spinal dorsal horn, and the SNI-dependent increase in p-cofilin was remarkably reversed by knockdown of NL1 in excitatory neurons. These results indicate that NL1 in excitatory neurons could regulate cofilin phosphorylation. Together, cofilin phosphorylated by NL1 in excitatory neurons might promote actin assembly, which facilitates spine/synapse formation, regulates spine/synaptic plasticity, and eventually leads to the development of neuropathic pain.

Past work has shown that post-operative pain increases the surface delivery of GluR1 in the dorsal horn of rats (Guo et al., 2014). GluR1 trafficking in dorsal horn neurons plays a pivotal role in inflammatory pain. Mice lacking GluR1 subunits show a loss of pain plasticity and a significant reduction in acute inflammatory hyperalgesia (Peng et al., 2012). The increase in the expression of GluR1 is related to type-2 diabetic neuropathic pain (Hartmann et al., 2004). They did not mention the role of GluR1 in pure neuropathic pain. We found that GluR1 was obviously increased in the dorsal horn of Vglut2-cre^+/–^ mice following SNI. Thus, GluR1 may be associated with the regulation of neuropathic pain.

Both AMPAR levels at synapses and AMPAR-dependent synaptic transmission are reduced in hippocampal slices of newborn NL1 knockout mice (Zhu et al., 2020). A unique intracellular tyrosine in NL1 regulates AMPAR recruitment during synapse differentiation and potentiation (Letellier et al., 2018). Synaptic potentiation is characterized by the insertion of AMPARs, which is a form of functional spine plasticity (Cingolani and Goda, 2008). GluR1 is a subtype of AMPAR. Our confocal images showed that GluR1 was co-localized with NL1 in excitatory neurons. Knockdown of NL1 in excitatory neurons blocked the increase of GluR1 caused by SNI. Therefore, we predict the mechanism by which NL1 in excitatory neurons participates in neuropathic pain involves GluR1-dependent synaptic enhancement, affecting synaptic plasticity of excitatory neurons.

Mounting evidence has confirmed that the enhanced synaptic plasticity of nociceptive interneurons in the superficial spinal dorsal horn is the basis of central sensitization in neuropathic pain (Kuner, 2015; Peirs and Seal, 2016; Alles and Smith, 2018; Zhang et al., 2019). Here, our study provides a possible mechanism for NL1-associated synaptic plasticity of excitatory interneurons in neuropathic pain. A reduction of NL1 in excitatory neurons inhibited the SNI-dependent increase in p-cofilin and GluR1 in Vglut2^+/–^ mice subjected to SNI, possibly leading to changes in synaptic plasticity of excitatory interneurons, which revealed a potentially specific role of NL1 in excitatory neurons in the development of neuropathic pain.

It should be acknowledged that there are some limitations to our study. Although we have observed that NL1 in excitatory neurons participated in the development of neuropathic pain, the potential regulatory mechanism is unclear. In addition to this, we only investigated the role of NL1 in excitatory neurons in neuropathic pain, and we have not explored the influence of NL1 in inhibitory neurons in nociceptive transmission. Moreover, we did not examine the influence of reducing the expression of p-cofilin in excitatory neurons or its effects on NL1 and GluR1 in the spinal dorsal horn. Indeed, further research is required to confirm our conclusions.

In summary, we demonstrated a mechanism for NL1-related synaptic plasticity of excitatory interneurons. We found that most excitatory neurons expressed NL1 and that the expression of NL1 was upregulated in Vglut2-cre^+/–^ mouse after SNI surgery. We also found that NL1 in excitatory neurons promoted the phosphorylation of cofilin and regulated the expression of GluR1 in the spinal dorsal horn of Vglut2-cre^+/–^ mice following SNI. Furthermore, knockdown of NL1 in excitatory neurons by an RNAi AAV reduced expression of p-cofilin and GluR1 and alleviated mechanical allodynia. We provide new molecular insight regarding the management of neuropathic pain.

## Conclusion

Based on the results of this study, upregulation of NL1 contributed to neuropathic pain, which may be involved in phosphorylation of cofilin and an increase in GluR1. Our findings suggest a new target to alleviate neuropathic pain.

## Data Availability Statement

The original contributions presented in the study are included in the article/supplementary material, further inquiries can be directed to the corresponding authors.

## Ethics Statement

The animal study was reviewed and approved by the Animal Use and Care Committee of Hubei University of Medicine (Shiyan, China).

## Author Contributions

XZ and CK conceived the project and designed the experiments. JO, XC, SS, and XL performed the experiments. JO, XX, and XY analyzed the data. JO wrote the manuscript under the guidance of CK and XZ. All authors contributed to the article and approved the submitted version.

## Conflict of Interest

The authors declare that the research was conducted in the absence of any commercial or financial relationships that could be construed as a potential conflict of interest.

## Abbreviations

AAV: adeno-associated virus
AMPAR: α -amino-3-hydroxy-5-methyl-4-isoxazolepropionic acid receptor
CTD: C-terminal domain
EGFP: enhanced green fluorescent protein
LIMK: LIM-domain protein kinase
NC-AAV: native control adeno-associated virus
NL1-AAV: recombinant RNAi adeno-associated virus targeting NL1
NL1: neuroligin 1
PBS: phosphate buffered saline
p-cofilin: phosphorylated cofilin
PSD: postsynaptic density
PWT: paw withdrawal threshold
SEM: standard error of the mean
SNI: spared nerve injury
WT: wild type.
